# A machine learning model for predicting obesity risk in patients with diabetes mellitus: analysis of NHANES 2007–2018

**DOI:** 10.3389/fpubh.2025.1606751

**Published:** 2025-08-22

**Authors:** Wenqiang Wang, Ruiqing Mo, Xingyu Chen, Sijie Yang

**Affiliations:** ^1^Department of Plastic and Reconstructive Surgery, The People’s Hospital of Guangxi Zhuang Autonomous Region & Research Center of Medical Sciences, Guangxi Academy of Medical Sciences, Nanning, Guangxi, China; ^2^Department of Bone and Joint Surgery, Guangxi Diabetic Foot Salvage Engineering Research Center, The First Affiliated Hospital of Guangxi Medical University, Nanning, China

**Keywords:** diabetes mellitus, obesity, machine learning, NHANES, LASSO regression, predictive modeling, nomogram

## Abstract

**Background:**

Obesity is a prevalent and clinically significant complication among individuals with diabetes mellitus (DM), contributing to increased cardiovascular risk, metabolic burden, and reduced quality of life. Despite its high prevalence, the risk factors for obesity within this population remain incompletely understood. With the growing availability of large-scale health datasets and advancements in machine learning, there is an opportunity to improve risk stratification. This study aimed to identify key predictors of obesity and develop a machine learning-based predictive model for patients with T2DM using data from the National Health and Nutrition Examination Survey (NHANES).

**Methods:**

Data from adults with diabetes were extracted from the NHANES 2007–2018 cycles. Participants were categorized into obese and non-obese groups based on BMI. Least absolute shrinkage and selection operator (LASSO) regression with 10-fold cross-validation was used to select relevant features. Subsequently, nine machine learning algorithms—including logistic regression, random forest (RF), radial support vector machine (RSVM), k-nearest neighbors (KNN), XGBoost, LightGBM, decision tree (DT), elastic net regression (ENet), and multilayer perceptron (MLP)—were employed to construct predictive models. Model performance was evaluated based on area under the ROC curve (AUC), calibration curves, Brier score, and decision curve analysis (DCA). The best-performing model was visualized using a nomogram to enhance clinical applicability.

**Results:**

A total of 3,794 participants with type 2 diabetes were included in the analysis, of whom 57.0% were classified as obese. LASSO regression identified 19 key variables associated with obesity. Among the nine machine learning models evaluated, the logistic regression model demonstrated the best overall performance, with the lowest Brier score. It also showed good discrimination (AUC = 0.751 in the training set and 0.781 in the test set), favorable calibration, and consistent clinical utility based on decision curve analysis (DCA). A nomogram was constructed based on the logistic regression model to facilitate individualized risk prediction, with total points corresponding to predicted probabilities of obesity.

**Conclusion:**

Obesity remains highly prevalent among individuals with type 2 diabetes. Our findings highlight key clinical features associated with obesity risk and provide a practical tool to aid in early identification and individualized management of high-risk patients.

## Background

Diabetes mellitus (DM) has become one of the most prevalent chronic metabolic diseases worldwide, with a significant increase in prevalence over the past few decades, particularly in low- and middle-income countries (1). According to the International Diabetes Federation (IDF), approximately 537 million adults globally were living with diabetes in 2021, and this number is projected to rise to 783 million by 2045 (2). Diabetes not only severely impacts patients’ quality of life but also imposes a substantial socioeconomic burden (3). Global healthcare costs related to diabetes and its associated complications amount to several hundred billion dollars annually, and this trend is expected to continue growing (4).

The occurrence of obesity in the DM patient group is significantly higher than in the non-diabetic population, which not only increases the risk of cardiovascular diseases, metabolic syndrome, and kidney diseases but also exacerbates the healthcare and social burden (5). However, even within the diabetic patient population, the incidence and severity of obesity show considerable variation (6). Research has shown that factors such as age, sex, race, lifestyle, dietary habits, and genetic predispositions can all influence the occurrence of obesity, and these complex factors make predicting the risk of obesity in diabetic patients more challenging (5).

In recent years, the rapid advancement of machine learning (ML) technology has provided new opportunities to address this issue, as sophisticated predictive models can effectively identify DM patients at high risk of obesity (7–9). The National Health and Nutrition Examination Survey (NHANES) offers high-quality, extensive clinical data, making it particularly well-suited for developing and validating predictive models. While previous studies have employed ML techniques to investigate the prediction of diabetes onset risk, treatment responses, and complications (such as cardiovascular diseases), there is relatively little research specifically focused on predicting the risk of obesity in diagnosed DM patients (10, 11).

The goal of this study is to utilize the NHANES database to develop a robust machine learning predictive model capable of distinguishing between DM patients at risk for obesity and those not at risk. Through a systematic analysis of variables, including demographic characteristics, clinical indicators, nutritional status, behavioral traits, and biochemical markers, this study aims to identify the key predictive factors for obesity in DM patients. Successful implementation of this predictive analysis will not only improve the effectiveness of personalized clinical interventions and patient outcomes but also significantly reduce the healthcare costs associated with obesity and diabetes. Ultimately, the results of this study will contribute to a deeper understanding of the mechanisms behind the occurrence of obesity in diabetes, providing practical and effective insights for clinical practice and healthcare policy development.

## Methods

### Study design and population

This study utilized cross-sectional data from the NHANES survey, collected in the United States between 2007 and 2018. The survey, conducted by the National Center for Health Statistics (NCHS) under the Centers for Disease Control and Prevention (CDC), aimed to provide a nationally representative assessment of the health and nutritional status of non-institutionalized civilians in the United States. Since NHANES is a publicly available database and has been approved by the Institutional Review Board (IRB) of NCHS, our institution confirmed that no additional ethical approval was required. Furthermore, the IRB acknowledges that NCHS adheres to strict ethical standards in its data collection and processing procedures, including obtaining informed consent from all participants and ensuring data anonymization. These measures guarantee full compliance with ethical guidelines for secondary data analysis.

### Assessment criteria for diabetes and obesity

**Figure 1:**
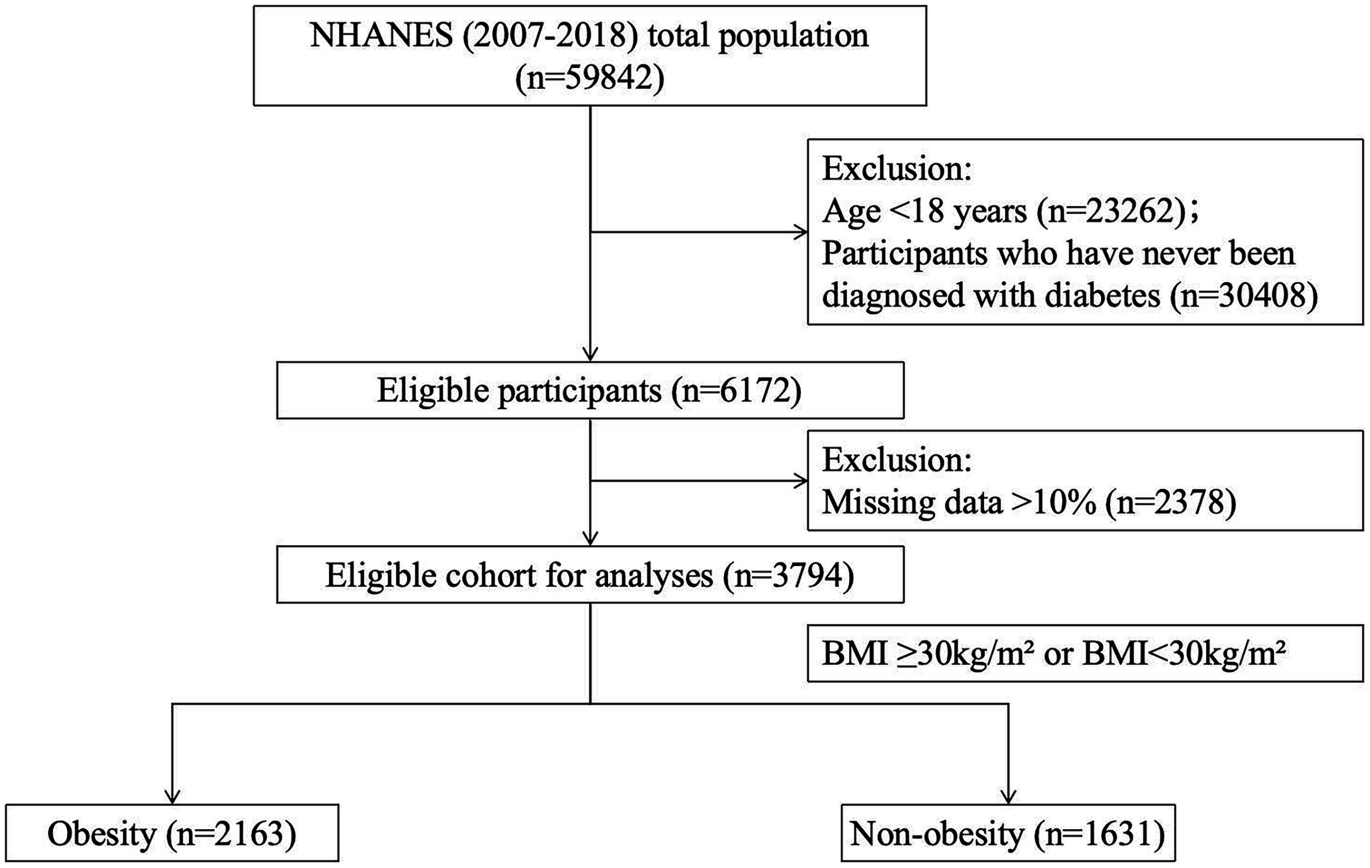
Flowchart of participant enrollment and exclusion.

Individuals diagnosed with diabetes were included in this study if they self-reported a diabetes diagnosis, had a fasting blood glucose level ≥126 mg/dL, had a hemoglobin A1c level ≥6.5%, or reported using anti-diabetic medications. Obesity was assessed based on participants’ body mass index (BMI), with a BMI ≥ 30.0 kg/m^2^ defining obesity, and those with a lower BMI classified as non-obese. To maintain data quality and consistency, records with more than 10% missing data were excluded, while multiple imputation was applied to records with minor missing values. Following these stringent inclusion and exclusion criteria, a total of 3,794 diabetes participants were included in the final analysis, consisting of 2,163 participants in the obesity group and 1,631 participants in the non-obesity group (Figure 1).

This study carefully selected a set of variables to investigate the factors associated with obesity in DM patients. These variables include demographic, socioeconomic, lifestyle, clinical, and biochemical factors. The demographic variables consist of gender, age, race, education level, and marital status. Education level is categorized as below high school, high school, and above high school. Marital status is classified into the following categories: married, widowed, divorced, separated, never married, or living with a partner. Race is divided into categories such as Mexican American, Other Hispanic, Non-Hispanic White, Non-Hispanic Black, and Other Race—including Multi-Racial. The Poverty-to-Income Ratio (PIR) is calculated by dividing household income by the corresponding poverty line for the survey year and location (12).

Lifestyle variables include smoking status (smoker or non-smoker) and alcohol consumption behavior (drinker or non-drinker). Physical activity levels are categorized as light, moderate, and vigorous exercise based on MET values.

Anthropometric measurements collected during the NHANES survey cycle include BMI. Biochemical markers include Alb, ALP, ALT, AST, TC, Scr, TG, UA, Hb, and HbA1c. Data on essential hypertension (EH), chronic heart disease (CHD), and depression were obtained through the questionnaire modules. Essential hypertension (EH) and coronary heart disease (CHD) were defined based on self-reported information from the “Blood Pressure and Cholesterol Questionnaire (BPQ)” and the Medical Conditions Questionnaire (MCQ) modules. Depressive symptoms were assessed using the PHQ-9 score, where a score of ≥10 indicates depression, and a score of <10 indicates no depression (13). Intake of total energy, protein, carbohydrate, total sugar, dietary fiber, total fat, vitamin B12, vitamin C, vitamin D, and vitamin K was obtained from the Dietary Data module.

### Statistical analysis

NHANES employs a complex multi-stage survey design to generate nationally representative data. Descriptive analysis involves calculating weighted averages with 95% confidence intervals and weighted percentages to account for the complex sampling design of the survey. Following NHANES analysis guidelines, chi-square tests were used for categorical variables, and weighted linear regression was applied to continuous variables to compare weighted groups. Unweighted data were used for model development and statistical analysis. Continuous variables are reported as means with standard deviations (SD), and categorical variables are presented as frequencies and percentages. Chi-square tests were used for categorical variables, and independent *t*-tests were applied to continuous variables to evaluate intergroup differences in clinical characteristics. A *p*-value of less than 0.05 was considered statistically significant, and all tests were two-tailed.

All statistical analyses in this study were performed using R software (version 4.2.2). Initially, LASSO regression with 10-fold cross-validation was conducted using the cv.glmnet() function from the glmnet package for variable selection. To enhance the robustness and comparability of model performance, we subsequently constructed and evaluated nine machine learning models: logistic regression (glm() with family = binomial), random forest (randomForest), radial support vector machine RSVM (e1071), k-nearest neighbors KNN (class), extreme gradient boosting XGBoost (xgboost), light gradient boosting machine LightGBM (lightgbm), decision tree DT (rpart), elastic net ENet (glmnet, *α* = 0.5), and multilayer perceptron MLP (nnet). Model performance was comprehensively assessed across four dimensions: (1) discrimination, measured by the area under the curve (AUC) using the pROC package; (2) calibration, evaluated via calibration plots using the rms and caret packages; (3) overall predictive accuracy, quantified using Brier scores calculated by the DescTools and ModelMetrics packages; and (4) clinical utility, determined by decision curve analysis (DCA) using the rmda package to estimate net clinical benefit at various threshold probabilities. All visualizations were generated using the ggplot2 package to ensure a clear and comprehensive presentation of the results. These methodological details have been incorporated into the revised Methods section to enhance clarity and reproducibility of the study.

### Model building process

LASSO regression and cross-validation were used for feature selection in the training set to identify significant predictive factors. Features with non-zero coefficients were retained for subsequent analysis. We randomly allocated 70% of the patient population to the training set and 30% to the testing set. Stratified sampling was applied to ensure a balanced distribution of the target variable between the two groups. To address the issue of class imbalance, the Synthetic Minority Over-sampling Technique (SMOTE) was used to generate a balanced dataset in the training set (14).

In this study, we employed nine machine learning algorithms, including logistic regression, to model and predict the risk of obesity among patients with diabetes. These algorithms comprised logistic regression, random forest (RF), radial support vector machine (RSVM), k-nearest neighbors (KNN), extreme gradient boosting (XGBoost), light gradient boosting machine (LightGBM), decision tree (DT), elastic net regression (ENet), and multilayer perceptron (MLP). Model construction was performed on the training set, and only after repeated training yielded stable results were the models evaluated on the independent testing set. Model performance was assessed across four dimensions: discrimination (measured by ROC curves and AUC), calibration (evaluated using calibration plots), overall predictive accuracy (assessed using the Brier score), and clinical utility (measured by DCA to estimate net clinical benefit across a range of threshold probabilities). The model with the best overall performance was ultimately selected for clinical visualization and interpretation.

The model’s performance was evaluated based on classification accuracy and robustness. After training, the model was validated using the test set. The model’s classification performance was assessed using the ROC curve and the AUC metric (15). ROC curve analysis in the training set provided insights into the model’s predictive capability, while the AUC value was used to evaluate its discriminative ability.

Furthermore, a nomogram was developed based on the logistic regression model, providing an intuitive and user-friendly tool for clinical use (16). The nomogram visually illustrates the relative contribution of each predictor to the obesity risk score. Each predictor is assigned a score proportional to its regression coefficient, and the total score corresponds to the predicted probability of obesity. A calibration curve was created to assess the predictive performance of the nomogram by comparing the predicted and observed probabilities of obesity. The discriminatory ability of the nomogram was evaluated using the AUC of the ROC curve. DCA was performed to evaluate the clinical utility of the nomogram by quantifying the net benefit at different threshold probabilities.

## Results

### Baseline characteristics

A total of 3,794 participants were included in this study, with an obesity prevalence rate of 57.01% (2,163/3,794). Among the participants, 1,990 were male and 1,804 were female, with average ages of 60.49 ± 12.66 years and 60.22 ± 12.96 years, respectively.

Compared to non-obese diabetic patients, those in the obese group were younger (57.85 ± 12.58 years vs. 63.69 ± 12.32 years), and the proportion of females was higher in the obese group (1,179/2,163 vs. 625/1,630). Additionally, significant differences were observed in education level, marital status, and race between the two groups. Participants in the obese group slept significantly less than those in the non-obese group (6.98 ± 1.67 vs. 7.26 ± 1.66, *p* < 0.05). Contrary to expectations, there were no significant differences in PIR, smoking, or drinking behaviors between the two groups (*p* > 0.05). Regarding past diseases, the obesity group had a higher prevalence of essential hypertension and depression (*p* < 0.05), while there were no significant differences in the prevalence of CHD and stroke (*p* > 0.05).

Regarding blood markers, significant differences were observed in ALB, ALP, ALT, Scr, TG, UA, and HbA1c between the two groups (*p* < 0.05). No differences were found in the levels of AST, TC, and Hb between the two groups (*p* > 0.05). In terms of energy intake, participants in the obesity group consumed more energy (1,964.70 ± 956.79 vs. 1,863.92 ± 854.06, *p* < 0.05). Significant differences were observed in the intake of carbohydrates, total sugar, dietary fiber, and total fat between the two groups (*p* < 0.05), while no differences were found in the intake of vitamin B12, C, and D (*p* > 0.05). Table 1 presents all the significant variables.

**Table 1:** Baseline data of the obese and non-obese groups [mean ± SD or *n* (%)].

Variable	Total	Obese	Non-obese	Statistic	*p*-value
*N*	3,794	2,163	1,630		
Sex				97.049	<0.001*
Male		984	1,006		
Female		1,179	625		
Age (years)		57.85 ± 12.58	63.69 ± 12.32	−14.329	<0.001*
Education				1878852.0	<0.001*
Below high school		648	563		
High school		500	398		
Above high school		1,015	670		
Marital status				23.558	<0.001*
Married		1,196	947		
Separated/widowed/divorced		625	511		
Never married		239	110		
Living with partner		103	63		
Race				166.116	<0.001*
Mexican American		393	277		
Other Hispanic		220	204		
Non-Hispanic White		833	540		
Non-Hispanic Black		605	329		
Other Race		112	281		
PIR		2.32 ± 1.55	2.31 ± 1.54	0.041	0.967
Drinking				1.894	0.169
No		703	495		
Yes		1,460	1,136		
Smoking				1.141	0.285
No		1,100	800		
Yes		1,063	831		
Activity level				5.45	0.066
Light		1,666	1,302		
Moderate		183	109		
Vigorous		314	220		
Sleep time (hours)		6.98 ± 1.67	7.26 ± 1.66	−5.234	<0.001*
EH				89.466	<0.001*
No		582	678		
Yes		1,581	953		
Depression					
No		1730	1,493	96.246	<0.001*
Yes		433	138		
CHD				1.308	0.253
No		1959	1,458		
Yes		204	173		
Stroke		Obesity	Non-obesity	0.0	1.000
No		1991	1,502		
Yes		172	129		
ALB (g/dL)		40.68 ± 3.37	42.00 ± 3.43	−11.826	<0.001*
ALP (U/L)		28.41 ± 19.39	24.48 ± 15.53	6.92	<0.001*
ALT (U/L)		27.01 ± 16.10	25.21 ± 12.36	3.908	<0.001*
AST (U/L)		76.89 ± 25.93	75.39 ± 27.76	1.692	0.091
TC (mg/dL)		4.82 ± 1.17	4.75 ± 1.22	1.884	0.060
Scr (mg/dL)		84.83 ± 41.24	89.34 ± 53.92	−2.818	0.005*
TG (mg/dL)		2.24 ± 1.56	2.06 ± 1.69	3.421	<0.001*
UA (mg/dL)		354.26 ± 95.46	328.58 ± 88.24	8.567	<0.001*
Hb (g/dL)		13.82 ± 1.60	13.88 ± 1.60	−1.176	0.240
HbA1c (%)		7.41 ± 1.70	7.29 ± 1.77	2.1	0.036*
Total energy (kcal)		1964.70 ± 956.79	1863.92 ± 854.06	3.416	<0.001*
Protein (g)		78.33 ± 40.86	75.04 ± 40.24	2.483	0.013*
Carbohydrate (g)		230.00 ± 117.46	222.18 ± 105.76	2.151	0.032*
Total sugar (g)		96.52 ± 70.88	88.49 ± 64.42	3.638	<0.001*
Dietary fiber (g)		16.01 ± 10.09	16.93 ± 10.79	−2.674	0.008*
Total fat (g)		79.11 ± 46.97	72.80 ± 42.39	4.328	<0.001*
Vitamin B12 (μg)		4.69 ± 5.74	4.64 ± 7.66	0.217	0.828
Vitamin C (mg)		75.68 ± 93.38	74.75 ± 79.97	0.331	0.741
Vitamin D (μg)		4.43 ± 6.18	4.30 ± 4.85	0.707	0.479
Vitamin K (μg)		105.73 ± 159.92	106.34 ± 160.07	−0.117	0.907

*p-value<0.05.

### Feature importance analysis

**Figure 2:**
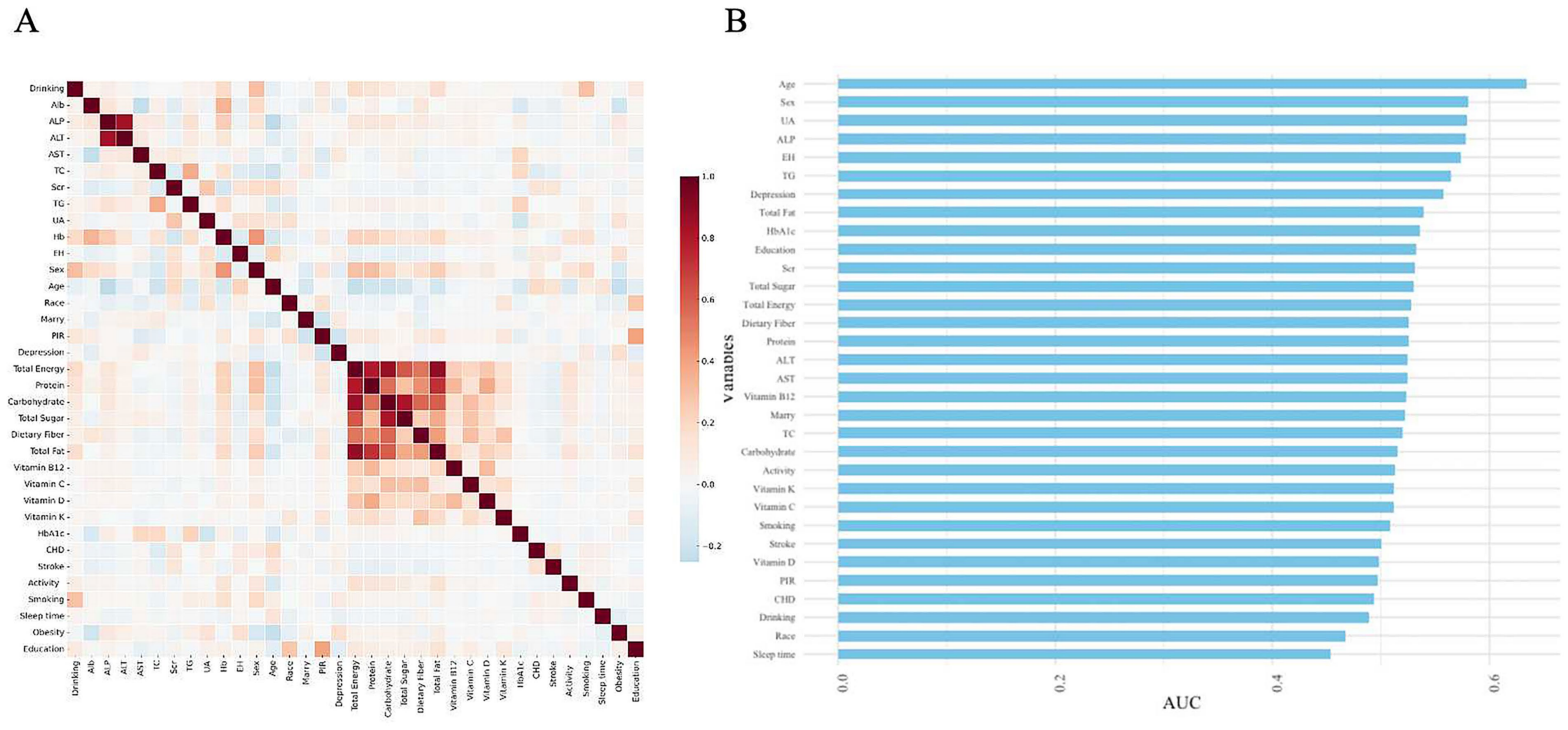
Heatmap of correlations between various variables and their predictive capabilities. **(A)** Heatmap of correlations. **(B)** Feature importance ranking of variables in the model based on AUC values.

In the variables of DM participants, the occurrence of obesity is associated with several factors (Figure 2A). Age appears to be the most significant variable, followed by gender and UA, suggesting that age, gender, and UA may play crucial roles in the obesity risk of diabetic patients. Furthermore, variables such as ALP, essential hypertension, TG, and depression showed significant predictive value. Other influencing factors include total fat, HbA1c, and education level, among others. In comparison, variables like smoking, alcohol consumption, vitamin intake, and sleep duration demonstrated relatively low predictive performance, indicating a weaker correlation with obesity risk (Figure 2B).

### Machine learning model comparison after LASSO selection

**Figure 3:**
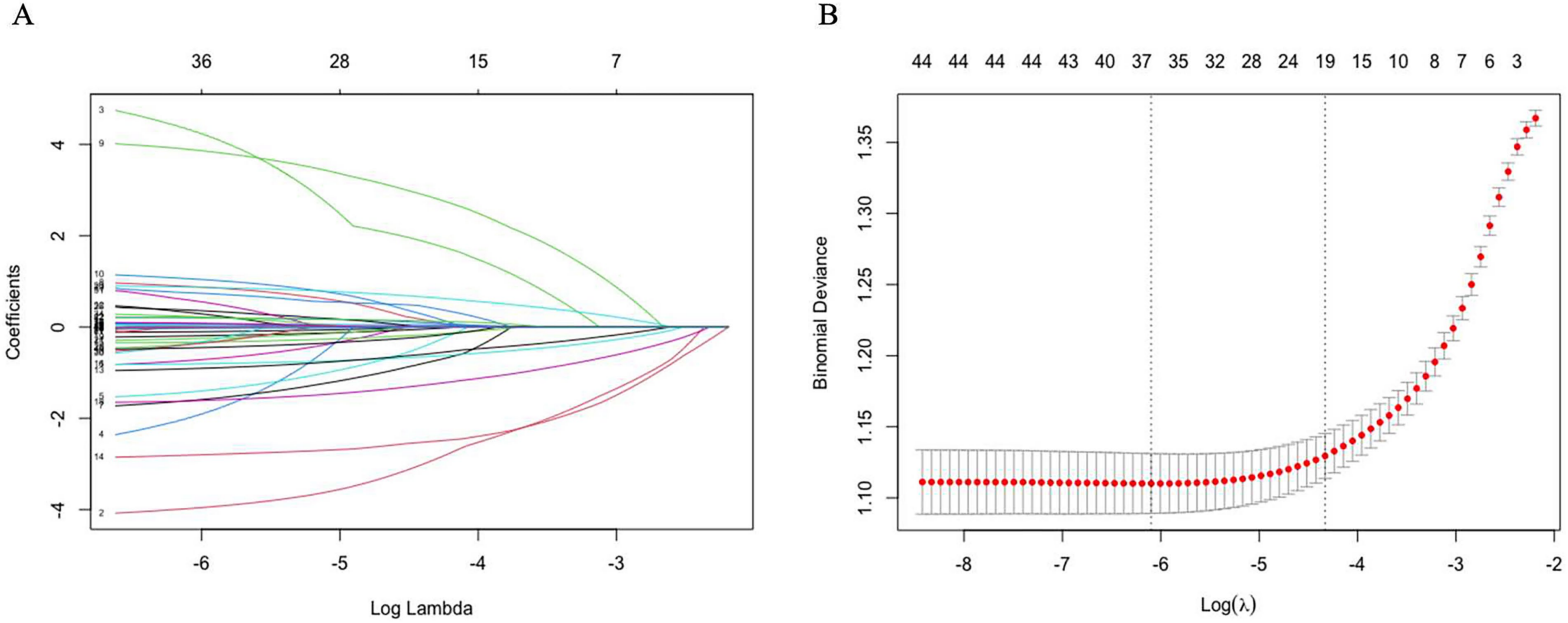
Coefficient trajectories and optimal lambda selection in LASSO regression. **(A)** LASSO coefficient path plot. **(B)** Least angle regression path plot. After using the Lasso regression analysis method, 19 variables were identified as key factors for diagnosing obesity.

To enhance the model’s robustness and minimize potential overfitting, LASSO regression was applied for the initial selection of predictor variables in the training set, with the optimal regularization parameter determined using 10-fold cross-validation (17). The LASSO model initially identified 19 variables with non-zero coefficients, including sex, age, race, marital status, PIR, ALB, ALP, AST, Scr, TG, UA, Hb, and sleep duration (Figure 3 and Supplementary Table S1).

**Figure 4:**
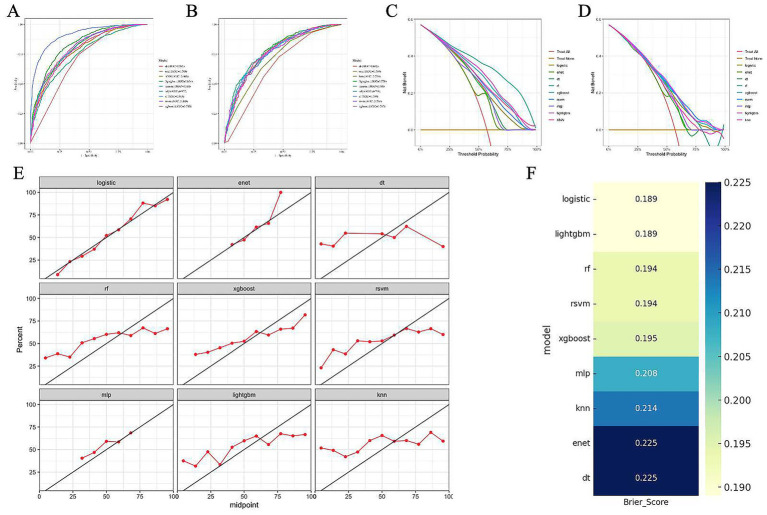
Performance evaluation of nine machine learning models for obesity prediction in patients with diabetes. **(A–B)** Receiver operating characteristic (ROC) curves of nine models in the training set **(A)** and test set **(B)**, respectively. The area under the curve (AUC) values are shown in the legend. **(C–D)** Decision curve analysis (DCA) of the models in the training set **(C)** and test set **(D)**, indicating net clinical benefit across a range of threshold probabilities. **(E)** Calibration plots of each model in the test set. The red line represents the observed probability, and the diagonal black line indicates perfect calibration. **(F)** Heatmap of Brier scores for each model in the test set. Lower Brier scores indicate better overall predictive accuracy.

Based on the selected variables, nine machine learning models were developed, including logistic regression, RF, RSVM, KNN, XGBoost, LightGBM, DT, ENet, and MLP. All models were stably trained on the training set and subsequently evaluated on the testing set. Among these, the logistic regression model demonstrated the best overall performance across multiple evaluation dimensions. It exhibited strong discriminatory power, with an AUC of 0.751 in the training set and 0.781 in the testing set, indicating reliable differentiation between obese and non-obese patients (Figures 4A,B). In terms of clinical applicability, DCA showed that the logistic model provided the greatest net benefit across a range of threshold probabilities (Figures 4C,D). The model also achieved favorable calibration, with predicted probabilities closely aligning with observed outcomes, and yielded the lowest Brier score (0.189), reflecting high overall predictive accuracy (Figures 4E,F). These findings support the selection of logistic regression as the optimal model for obesity risk prediction in patients with diabetes. Accordingly, logistic regression was selected as the optimal predictive model in this study.

### Construction and visualization of the logistic regression model

**Figure 5:**
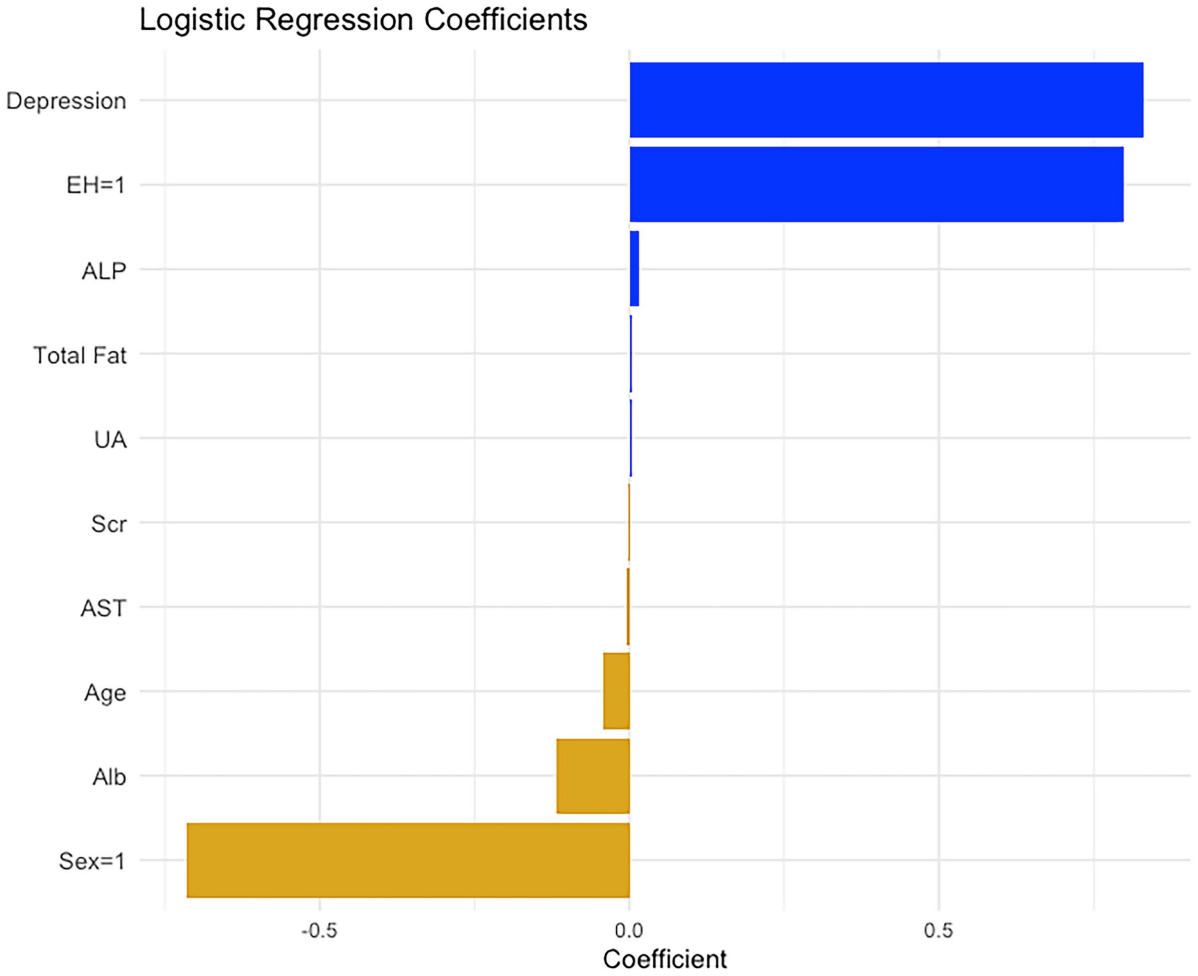
The regression coefficients of the 10 significant variables after logistic regression analysis. These regression coefficients are used to construct the nomogram for diagnosing diabetic obesity.

Following the identification of logistic regression as the optimal predictive model among the nine machine learning algorithms, we further visualized the model coefficients and constructed a clinically applicable prediction tool. As shown in Figure 5, among the variables included in the logistic model, depression and EH emerged as the strongest positive predictors, suggesting that patients with these conditions are at increased risk of obesity. Additionally, metabolic indicators such as ALP and total fat intake also contributed positively. In contrast, variables such as male sex, Alb, and age were negatively associated with obesity risk, indicating a potential protective effect.

**Figure 6:**
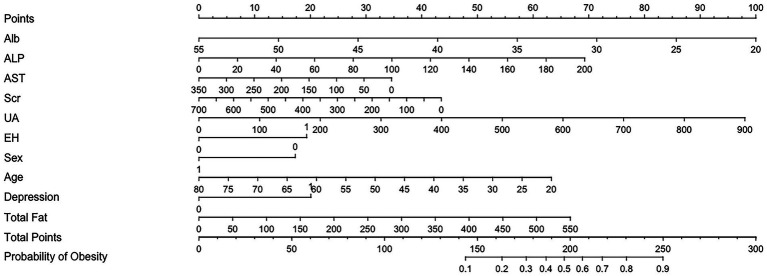
Nomogram to estimate the risk of obesity in DM patients. The points assigned to each predictor are summed to obtain the total score. A vertical line drawn from the total score corresponds to the predicted probability of obesity.

Based on the estimated regression coefficients, a nomogram was constructed to facilitate individualized risk assessment (Figure 6). This nomogram incorporates all significant predictors from the model. By locating the value of each patient’s characteristic on the corresponding axis, assigning points, and summing the total score, clinicians can estimate the predicted probability of obesity. The tool is intuitive, easy to interpret, and clinically applicable, offering a practical method to support early identification and personalized management of obesity risk in patients with diabetes.

**Figure 7:**
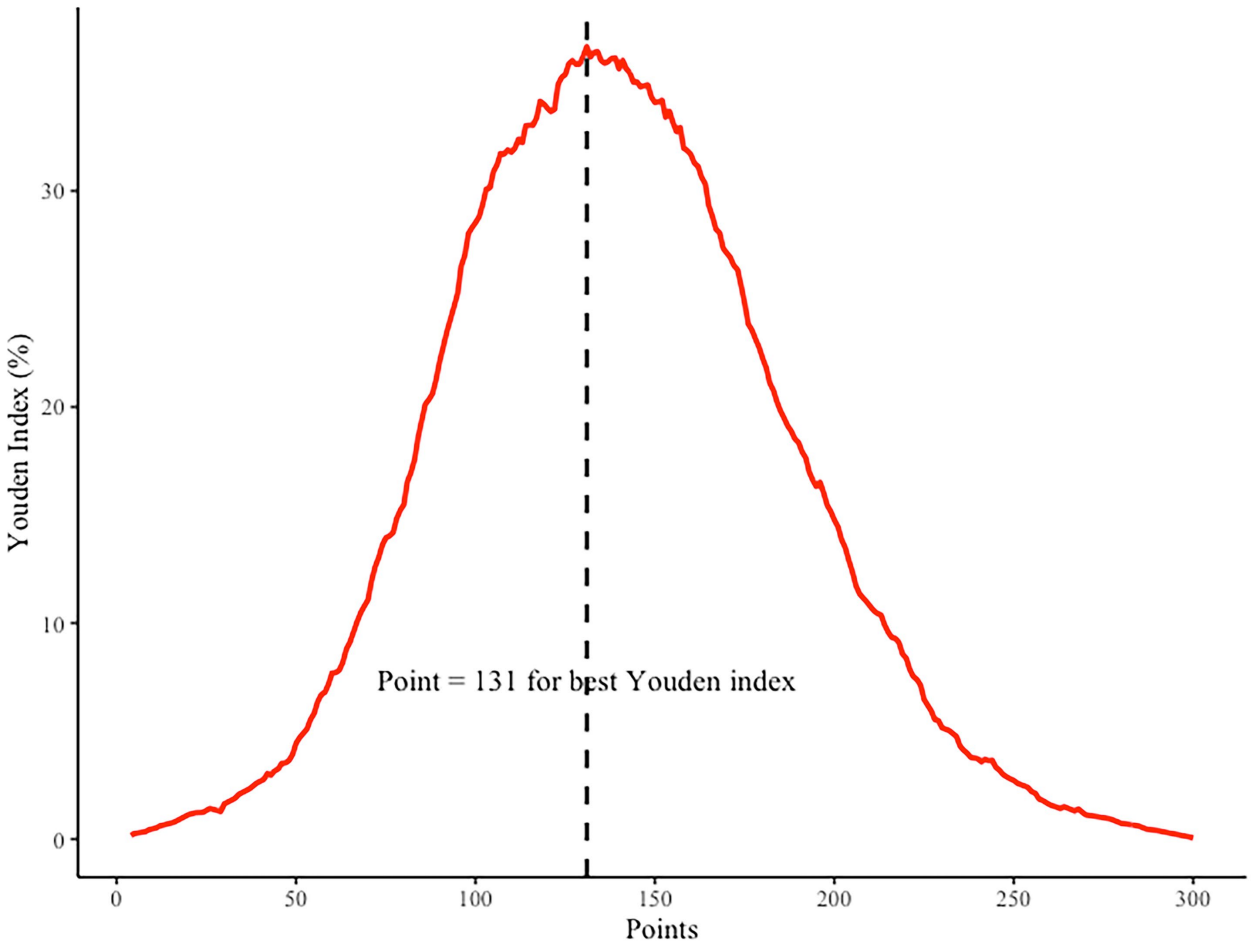
Youden index analysis for determining the optimal cutoff point of the nomogram. The highest Youden index corresponds to a total point score of 131, as indicated by the dashed vertical line.

To further enhance the clinical interpretability of the nomogram, we determined the optimal cutoff point based on the maximum Youden index. As shown in Figure 7, the Youden index peaked when the total nomogram points reached 131, indicating the most balanced trade-off between sensitivity and specificity at this threshold. This cutoff value can serve as a reference point in clinical decision-making, enabling physicians to identify high-risk patients who may benefit from early lifestyle intervention or closer metabolic monitoring (Table 2).

**Table 2:** Odds ratios and 95% confidence intervals from multivariate logistic regression analysis.

Variable	OR	2.5%	97.5%
Alb	0.8885973	0.863925	0.9139741
ALP	1.0144196	1.0085186	1.0203552
AST	0.9959228	0.9925759	0.9992811
Scr	0.9974336	0.9954171	0.9994542
UA	1.0045131	1.0034784	1.0055488
EH	2.2246476	1.8358482	2.6957877
Sex	0.4893815	0.4046678	0.5918293
Age	0.9573351	0.9499819	0.9647452
Depression	2.2946446	1.7645954	2.98391
Total fat	1.0050249	1.0028926	1.0071617

## Discussion

This study is based on data from the NHANES 2007–2018, involving a retrospective analysis of nearly 3,800 diabetic patients. The aim was to identify which indicators are particularly significant in the context of obesity among various clinical and lifestyle factors. Using machine learning algorithms, we ultimately identified 10 variables, including traditional physiological indicators such as ALP, Scr, and AST, as well as less frequently discussed variables, such as depression. This mixed result highlights an important issue: diabetes combined with obesity is not solely due to excess calorie intake, but rather a complex process involving metabolic, emotional, behavioral, and organ function imbalances.

This study demonstrates a certain degree of innovation in terms of research perspective, methodological design, and the significance of the findings. It focuses on predicting the risk of obesity specifically within the population of individuals with diabetes. Although this topic has been discussed in the existing literature, systematic predictive modeling targeted at this specific subgroup remains relatively limited. Our study seeks to contribute additional insight in this underexplored area. Methodologically, we applied LASSO regression for variable selection and developed predictive models using nine commonly used machine learning algorithms. Model performance was comprehensively evaluated across multiple dimensions, including AUC, calibration (calibration plots), overall predictive accuracy (Brier score), and clinical utility (DCA). Furthermore, we employed nomogram construction to enhance model interpretability and clinical applicability. In terms of results, the study identified several key variables strongly associated with obesity and established a predictive model with promising performance and generalizability. These findings may serve as a theoretical basis and practical reference for the early identification and individualized intervention of obesity risk in patients with diabetes.

First, it is important to highlight the prominent role of psychological factors, particularly depression, in the model. In the past, we tended to attribute obesity and diabetes to “eating too much and moving too little,” but the inclusion of depression as a variable in the model reminds us that we cannot overlook the long-term effects of psychological states on energy metabolism and behavior patterns. Existing studies suggest that chronic depression may disrupt appetite regulation mechanisms through abnormal activation of the HPA axis, which in turn influences eating preferences, sleep patterns, and even the willingness to exercise (18, 19). This impact may be more pronounced in diabetic patients. At the same time, the inclusion of traditional factors such as hypertension, age, and gender is not unexpected. On one hand, these serve as “background variables” for disease progression, and on the other hand, they provide the empirical basis for clinical judgment. Therefore, we should pay more attention to whether there are factors, beyond the “familiar” variables, that we have previously overlooked, which may be subtly altering the course of the disease.

Some of the findings related to biochemical markers are quite thought-provoking. The appearance of ALP, AST, and Scr suggests that the mechanisms underlying diabetes combined with obesity may have far exceeded our conventional understanding of “glucose metabolism disorders.” ALP and AST are typically regarded as indicators of liver function, and their changes may already signal the presence of visceral fat accumulation, fatty liver disease, or even inflammation in the asymptomatic stage (20). This could explain why, in recent years, NAFLD has been recognized as a precursor to “novel liver-derived diabetes” (21). Scr, being a relatively stable marker of kidney function, is unsurprising in this population. Diabetes is a major contributor to kidney damage, and the symptoms of obesity further exacerbate the high-pressure burden on the glomerulus (22). More importantly, these markers are less likely to fluctuate compared to lifestyle variables, and as indicators of end-organ damage, they often provide a more accurate reflection of disease progression.

In terms of diet, the inclusion of total fat intake signals that “what” we eat may be more important than “how much” we eat. Although total sugar intake was not included in the final model, this contrasts with some findings in the literature. Possible explanations include: first, diabetic patients generally have an awareness of blood sugar control, which narrows the differences between groups; second, compared to carbohydrates, the metabolic effects of fat are more long-lasting and have a more direct impact on insulin resistance. Additionally, the presence of uric acid and albumin further supports the potential involvement of inflammation and oxidative stress in the pathology of obesity, a direction that has gained widespread attention in recent years (23). In general, the significance of these biochemical markers lies not only in their role as static diagnostic tools but also in their potential as dynamic warning signals, providing important information before clear metabolic imbalance is observed.

Naturally, it is somewhat surprising that some factors traditionally thought to be closely related to obesity, such as smoking, drinking, stroke history, physical activity, and sleep duration, did not enter our model. This does not necessarily imply that they are unimportant, but rather suggests that their explanatory power in the context of diabetes has been overshadowed by other factors. The metabolic effects of smoking and drinking may primarily manifest through cardiovascular and inflammatory pathways, rather than directly contributing to obesity (24). Physical activity and sleep data in NHANES are primarily based on self-reported questionnaires, which are susceptible to cognitive bias. Furthermore, these factors may exhibit collinearity with the selected variables, and weaker variables are more easily “pushed out of the model” in LASSO regression. In other words, the LASSO results are more about selecting variables that maintain independent explanatory power in high-risk populations such as “diabetes + obesity,” rather than simply listing all potential influencing factors.

Overall, our goal is not to create a comprehensive obesity prediction system, but rather to use a feature selection method from the machine learning field—LASSO-logistic regression—to identify a set of variables that truly possess independent explanatory power and predictive value, amidst numerous variables and highly redundant information. Compared to traditional regression, this machine learning method offers stronger dimensionality reduction capabilities and superior recognition of collinear variables, making the model more concise and stable, and thus suitable for clinical decision-making scenarios based on real-world data (25). It is noteworthy that we chose not to rely on complex “black-box” models, but instead employed a linear regularization algorithm with good interpretability, striking a balance between statistical significance and clinical applicability. The final results reveal that the selected variables span multiple dimensions, including psychology, biochemistry, nutrition, and organ function, which, to some extent, outline the biological feature spectrum of diabetes combined with obesity. This also suggests that in future chronic disease research and management, machine learning is not only a tool but also a perspective—it can help us unravel complex data and identify the key aspects that truly warrant attention and intervention.

### Limitation

This study has several limitations that should be acknowledged. First, the data were derived from the NHANES database between 2007 and 2018, and therefore may not fully capture more recent trends or behavioral and physiological changes in the post-COVID-19 era. Second, due to the cross-sectional nature of the NHANES data, causal inferences cannot be drawn from the observed associations. Third, although multiple machine learning algorithms were used and their performance compared, external validation using independent datasets was not conducted, which may limit the generalizability of the models. Fourth, some relevant variables such as genetic, environmental, or medication-related factors were not available in the dataset, potentially affecting the model’s completeness. Finally, although the study applied nomogram visualization, the clinical applicability of the prediction model should be further tested in prospective studies and real-world settings.

## Conclusion

In summary, this study identifies key risk factors associated with obesity among individuals with type 2 diabetes using large-scale population-based data and a comparative machine learning framework. Our findings underscore the multifactorial nature of metabolic dysregulation in this population, involving psychological, nutritional, biochemical, and organ function indicators. Among the nine models evaluated, logistic regression demonstrated the most balanced predictive performance and was used to construct a clinically interpretable nomogram. This tool may support early risk stratification and personalized intervention in diabetic patients. Future studies should focus on external validation and longitudinal tracking to enhance model generalizability and translational potential.

## Data Availability

The original contributions presented in the study are included in the article/Supplementary material, further inquiries can be directed to the corresponding authors.
